# Conditional Regulation of Blood Pressure in Response to Emotional Stimuli by the Central Nucleus of the Amygdala in Rats

**DOI:** 10.3389/fphys.2022.820112

**Published:** 2022-06-01

**Authors:** Ko Yamanaka, Hidefumi Waki

**Affiliations:** ^1^ Department of Physiology, Graduate School of Health and Sports Science, Juntendo University, Inzai, Japan; ^2^ Institute of Health and Sports Science and Medicine, Juntendo University, Inzai, Japan

**Keywords:** blood pressure, heart rate, licking behavior, classical conditioning, amygdala, rat, brain

## Abstract

Humans and animals can determine whether a situation is favorable to them and act accordingly. For this, the autonomic tuning of the cardiovascular system to supply energy to active skeletal muscles through the circulatory system is as important as motor control. However, how the autonomic cardiovascular responses are regulated in dynamically changing environments and the neuronal mechanisms underlying these responses remain unclear. To resolve these issues, we recorded the blood pressure and heart rate of head-restrained rats during dynamically changing appetitive and aversive classical conditioning tasks. The rats displayed various associations between conditioned stimuli and unconditioned stimuli in appetitive (sucrose water), neutral (no outcome), and aversive (air puff) blocks. The blood pressure and heart rate in the appetitive block gradually increased in response to the reward-predicting cue and the response to the actual reward vigorously increased. The reward-predictive response was significantly higher than the responses obtained in the neutral and aversive condition blocks. To investigate whether the reward-predictive pressor response was caused by orofacial movements such as anticipatory licking behavior, we separately analyzed high- and low-licking trials. The conditioned pressor response was observed even in trials with low-licking behaviors. Blood pressure and heart rate responses to the air puff-predicting cue in the aversive block were not significantly different from the responses in the neutral block. The conditioned blood pressure response rapidly changed with condition block switching. Furthermore, to examine the contribution of the amygdala as an emotion center to these conditioned responses, we bilaterally microinjected a GABA_A_ receptor agonist, muscimol, into the central nucleus of the amygdala. Pharmacological inactivation of the central nucleus of the amygdala significantly decreased the reward-predictive pressor responses. These results suggest that the blood pressure is adaptively and rapidly regulated by emotional conditioned stimuli and that the central nucleus of the amygdala participates in regulating the pressor response in dynamically changing situations.

## Introduction

In a dynamically changing environment, the ability to predict outcomes of future events allows getting rewards ahead of competitors and escaping from danger. The amygdala is classically believed to play a central role in negative emotions (Hilton and Zbrozyna, 1963; Pascoe and Kapp, 1985); however, its involvement in the emotional, attentional, and learning processes of external stimuli with positive consequences has also been reported in rodents (Holland and Gallagher, 1999; Everitt et al., 2003; Calu et al., 2010; Roesch et al., 2012; Kim et al., 2016), macaques (Baxter and Murray, 2002; Paton et al., 2006), and humans (Phelps and LeDoux, 2005). Shabel and Janak showed that the amygdala neuronal activities triggered by appetitive and aversive conditioned stimuli are similar and correlate with autonomic arousal such as changes in the blood pressure, suggesting that emotional arousal is coded in this brain region (Shabel and Janak, 2009; Shabel et al., 2011). Our previous study showed that electrical and chemical stimulations of the central nucleus of the amygdala (CeA) in anesthetized rats induce bidirectional (facilitatory or inhibitory) cardiovascular responses in a region-specific manner, indicating that the amygdala contributes to the neuronal circuitry modulating autonomic responses (Yamanaka et al., 2018). Conditioned cardiovascular responses are classically recorded during the anticipation of either appetitive or aversive outcomes (Harris and Brady, 1974; Cohen and Obrist, 1975). Because many of these studies have been conducted on free-moving animals, it is difficult to control animal behaviors such as freezing and fleeing. Furthermore, the autonomic cardiovascular response is dynamically regulated in subjects facing an environment where the emotional context dynamically changes. However, the amygdala’s involvement has not been demonstrated.

Here, we hypothesized that the amygdala plays a role in the autonomic cardiovascular regulation involved in emotional arousal in response to salient external stimuli. Therefore, we recorded the blood pressure and heart rate of head-restrained rats during dynamically changing appetitive and aversive classical conditioning tasks and examined the effects of amygdala manipulation.

## Materials and Methods

### Animals

A total of 10 male Wistar–Kyoto rats (age: 7 weeks and weight: 238 ± 39 g at the time of the first surgery) were used (Japan SLC, Inc., Japan). The animals were housed in a temperature-controlled room under a fixed 12/12 h dark/light cycle (6:00–18:00/18:00–6:00). Food was available in the home cage *ad libitum*. Water access was restricted during behavioral learning of the task (up to 24 h) to increase the motivation for sucrose rewards. Rat body weights were measured every day to control that they were not less than 85% of the original weight. A few agar blocks (containing 15 ml water) were regularly given in the home cages. The animals had access to water *ad libitum* after surgery recovery and on the weekends. All experiments were approved by the Ethics Committee for Animal Experiments at Juntendo University and complied with the guidelines set by the Japan Physiological Society.

### Surgeries

Two distinct surgeries were performed to implant first a radio transmitter for blood pressure recording and then head plates to fix the animal body during experiments. The recovery time after each surgery was more than a week. During surgery, the rectal temperature was monitored and maintained at 37°C using a heating pad (BWT-100, Bio Research Center, Japan). After surgery, antibiotics (benzylpenicillin, 1000 U, intramuscular, Meiji Seika Pharma, Japan) and analgesics (meloxicam, 1 mg/kg, subcutaneous, Boehringer Ingelheim, Germany) were administered.

### Implantation of a Transmitter for Telemetry

A telemetric radio transmitter (HD-S10; Data Sciences International, USA) for chronic blood pressure recording from the abdominal aorta was implanted, as described in previous studies (Waki et al., 2003; Yamanaka et al., 2018). Rats were anesthetized with pentobarbital sodium (50 mg/kg) by intraperitoneal (i.p.) administration and isoflurane (2.0%–2.5% for maintenance) using an inhalation anesthesia apparatus (Univentor 400 anesthesia unit, Univentor, Sweden). The level of anesthesia was checked by assessing limb withdrawal to noxious pinching. After an abdominal midline incision was made in rats in a supine position, the intestines were moved aside to allow visualization and isolation of the abdominal aorta. The aorta was temporary aortic occluded using a sterilized string to prevent severe blood loss, and the tip of the transmitter catheter was inserted into the abdominal aorta with a 21G needle guide. The transmitter catheter was then fixed using tissue adhesive (Vetbond, 3M, United States) and the transmitter was sutured to the ventral wall of the abdominal cavity.

### Implantation of a Head Plate

All procedures for the head plate implantation (CFR-1, Narishige, Japan) were previously established (Kimura et al., 2012). Rats were anesthetized with isoflurane (Pfizer, USA), 4.5%–5.0% for induction and 2.0%–2.5% for maintenance. Then, they were placed on a stereotaxic frame (SR-10R-HT, Narishige, Japan). A stainless-steel head plate was attached to the skull using tiny stainless-steel screw bolts (M1, 2 mm long; Yahata Neji Corporation, Japan) as anchors and dental resin cement (Super-Bond C&B, Sun Medical; Unifast II, GC Corporation, Japan).

### Dynamically Changing Classical Conditioning Tasks

**FIGURE 1:**
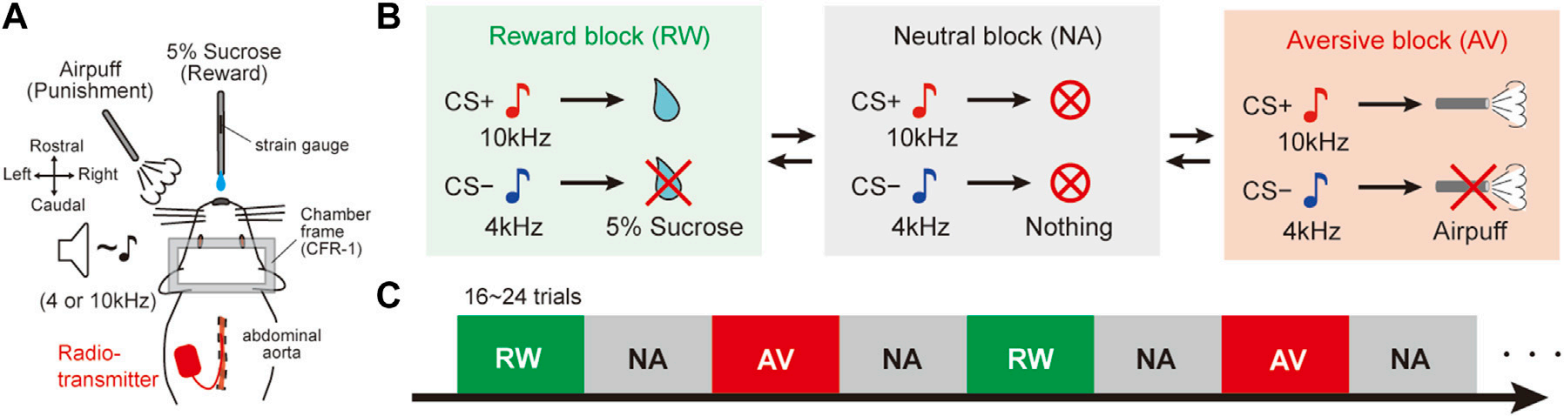
Dynamically changing classical conditioning task with emotional valence. **(A)** Schematic diagram of the behavioral task. Stainless tubes for reward and air-puff delivery were placed in front of the face of head-fixed rats. A radio transmitter was implanted into the abdominal aorta to record the blood pressure by telemetry. A strain gauge was attached to the reward tube to monitor the amplitude of licking movements. **(B)** Pavlovian procedure with three distinct context blocks. In the reward (RW) block, a tone cue (CS+, 10 kHz) preceded the reward (US+, 5% sucrose), and another tone (CS−, 4 kHz) was not followed by a reward (US−). In the aversive (AV) blocks, CS + preceded an air puff (US+) and no air puff (US−) followed CS−. Both CS tones preceded nothing in the neutral (NA) blocks. CS, conditioned stimulus; US, unconditioned stimulus. **(C)** Trial sequence of the behavioral task. NA blocks were alternately deployed between RW and AV blocks. There were 16–24 trials per block. The order of the blocks was fixed, but they randomly started with RW or AV every experimental day. There was no cue indicating the block switch.

After at least a week of post-surgery recovery, the behavioral investigation was initiated. The rats were trained using a behavioral testing system for a classical conditioning task (Task Forcer, O’Hara and Co., Ltd., Japan). This task was used as a behavioral model to evaluate autonomic responses during dynamic changing cue-outcome associations. Animals were fixed on a stereotaxic frame (SR-10R-HT, Narishige, Japan) in a sound-attenuated box (SAC-4201W, O’Hara and Co., Ltd., Japan). The beginning of the first session was dedicated to acclimatizing the rats to head fixation and teaching them that a reward was delivered from a spout tube located in front of their mouth. To achieve this aim, the rats were immobilized for 1 hour and given the reward several times until they licked the spout tube. Then, the task session started. Animals learned various associations between conditioned stimuli (CS) and unconditioned stimuli (US) in three experimental blocks (Figures 1A,B): 1) appetitive reward (RW) block: one tone cue (reward CS+, 10 kHz, 1 s, 60–70 dB) associated with sucrose delivery (reward US+; 5% sucrose, 0.08 ml) and another tone cue (reward CS−, 4 kHz, 1 s, 60–70 dB) associated with no reward (reward US−), 2) aversive (AV) block: the CS+ was associated with an air puff (aversive US+; 30–40 psi, 1 s) and the CS− was associated with no air puff (aversive US−), and 3) neutral (NA) block: both CS tones predicted nothing (neutral CS+ and CS−). The air puff was delivered through a stainless-steel tube (AG45-2–100, Φ2×100 mm, Kurita Seisakusho, Japan) placed 8–10 cm away from the rat’s face, below its left eye. The time interval between CS offset to US onset (CS–US interval) was 15 s. The inter-trial interval was 60 ± 15 s. Each block consisted of 16–24 trials, and the order of the trials (CS + or CS− trial) was pseudo-randomly assigned. The animals could not predict the timing of the block switch because of the lack of cue at block changing. The order of the block presentation was fixed as RW and AV blocks were alternatively presented and the NA block was intercalated between them (Figure 1C; RW → NA → AV → NA → RW →…). The daily trials randomly started with RW or AV. For the learning process, the rats were initially trained only in the RW block. The AV context was then added, and the NA context training was included last. Orofacial licking movements indicating reward anticipation and consumption were monitored by measuring the strain on the reward spout tube (Figure 1A; KFG-2N-120-C1-23 amplified by DPM-911B, Kyowa, Japan).

### Muscimol Injections

The link between context-based blood pressure responses and CeA activity was assessed using pharmacological inactivation experiments. Seven of the 10 animals were used in this experiment. The day before the injection experiment, two holes were drilled in the skull, approximately above the CeA, under anesthesia with isoflurane (2.0%–2.5% for maintenance) and covered with silicone (Dent Silicone-V, Shofu, Japan). Micropipettes were connected to a Hamilton microsyringe mounted on a syringe pump (LEGATO110, KD Scientific, United States) to control the injection rate (500 nL/min). The silicone lid was removed immediately before the classical conditioning task and a GABA_A_ receptor agonist (muscimol, 80 pmol, 100 nL, M1523-10 MG, Sigma-Aldrich, United States) was microinjected (Stotz-Potter et al., 1996) into the bilateral CeA (1.8 mm caudal, 3.0 mm lateral from the bregma, and 7.0 mm ventral from the dura) using a glass micropipette (an outside diameter of 20–30 μm; GC200F-10, Harvard Apparatus, United States) under local anesthesia with lidocaine (Xylocaine Polyamp 1%, AstraZeneca). In the control experiment, saline (100 nL, Otsuka, Japan) was injected to measure cardiovascular responses induced by the volume of liquid injected. The order of administration of muscimol and saline was alternated between the animals. After bilateral injections, the micropipette was pulled out and the behavioral task was started. After completing the final experiments, the chemical inactivation site was identified and controlled by injecting fluorescent microspheres (FluoSpheres, 100 nL, Thermo Fisher Scientific, United States) with a separate pipette at the same stereotaxic coordinates as those used for muscimol injections.

### Histology

After the last behavioral experiment, the rats were deeply anesthetized with sodium pentobarbital and isoflurane and intracardially perfused with saline and then 4% paraformaldehyde (163-20145, FUJIFILM Wako Pure Chemical Corporation, Japan). The brains were removed, post-fixed for at least 48 h in 4% paraformaldehyde, and then immersed in 30% sucrose. Once settled at the bottom of the sucrose solution, the brains were sliced into 50-μm-thick serial sections using a freezing microtome (REM-710; Yamato Kohki Industrial, Japan). The sections were then mounted on slides and imaged using a fluorescence microscope (EVOS FL Auto 2 imaging system, Thermo Fisher, United States) to map the drug injection sites in the amygdala.

### Data Analysis and Statistics

**FIGURE 2:**
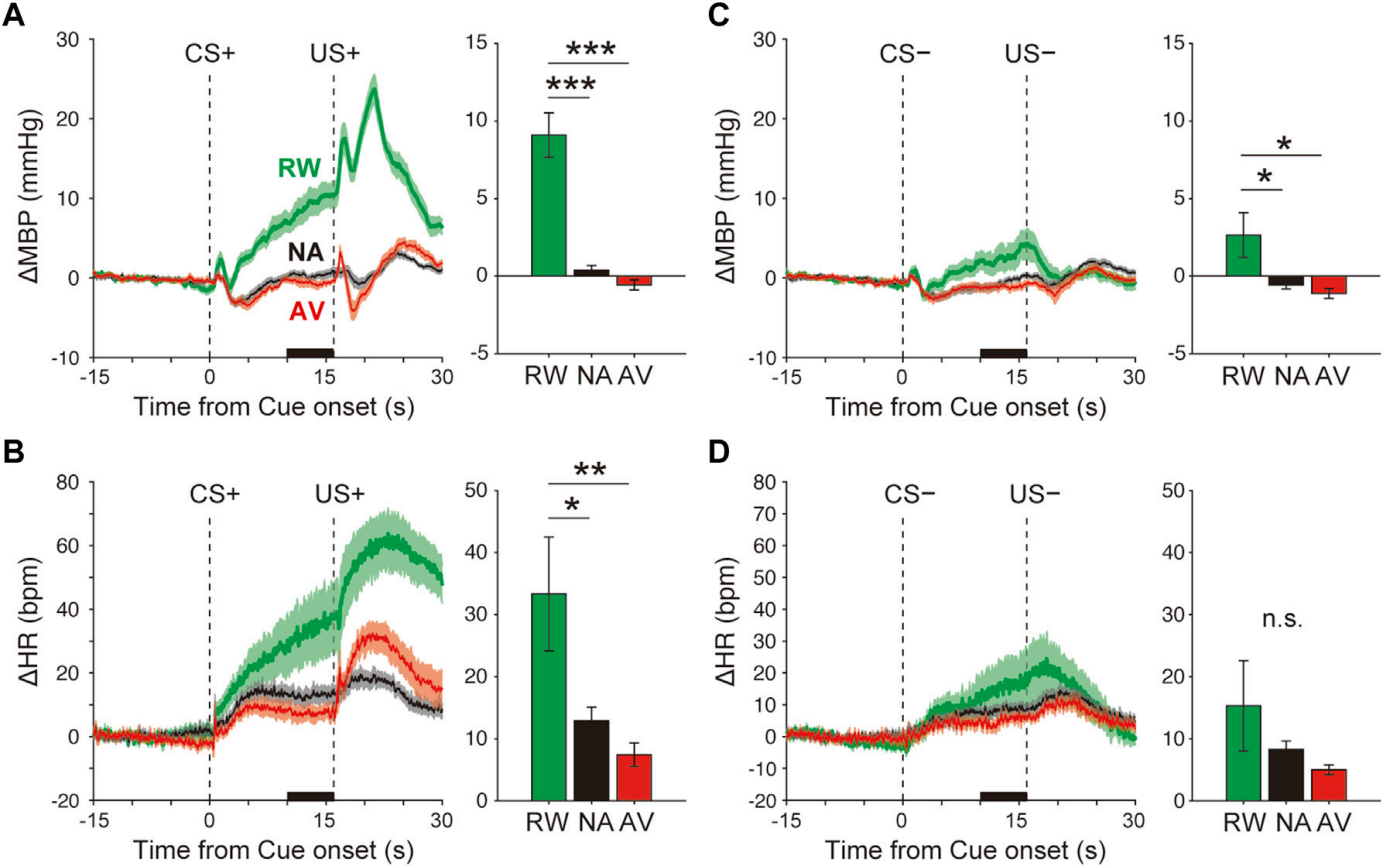
Blood pressure and heart rate responses during the Pavlovian conditioning task. **(A–B)** Average responses of ΔMBP **(A)** and ΔHR **(B)** to CS+ and US+ during the reward (RW; green line and bar), neutral (NA; black line and bar), and aversive (AV; red line and bar) condition blocks. Lines and shadows indicate the mean ± standard error. The thick black line on the *X*-axis indicates the time window for a quantitative analysis. **(C–D)** Same as **A**–**B**, but during CS− and US−. *p < 0.05, ***p < 0.001, one-way analysis of variance with a Tukey–Kramer post hoc test, and n = 10 rats.

**FIGURE 3:**
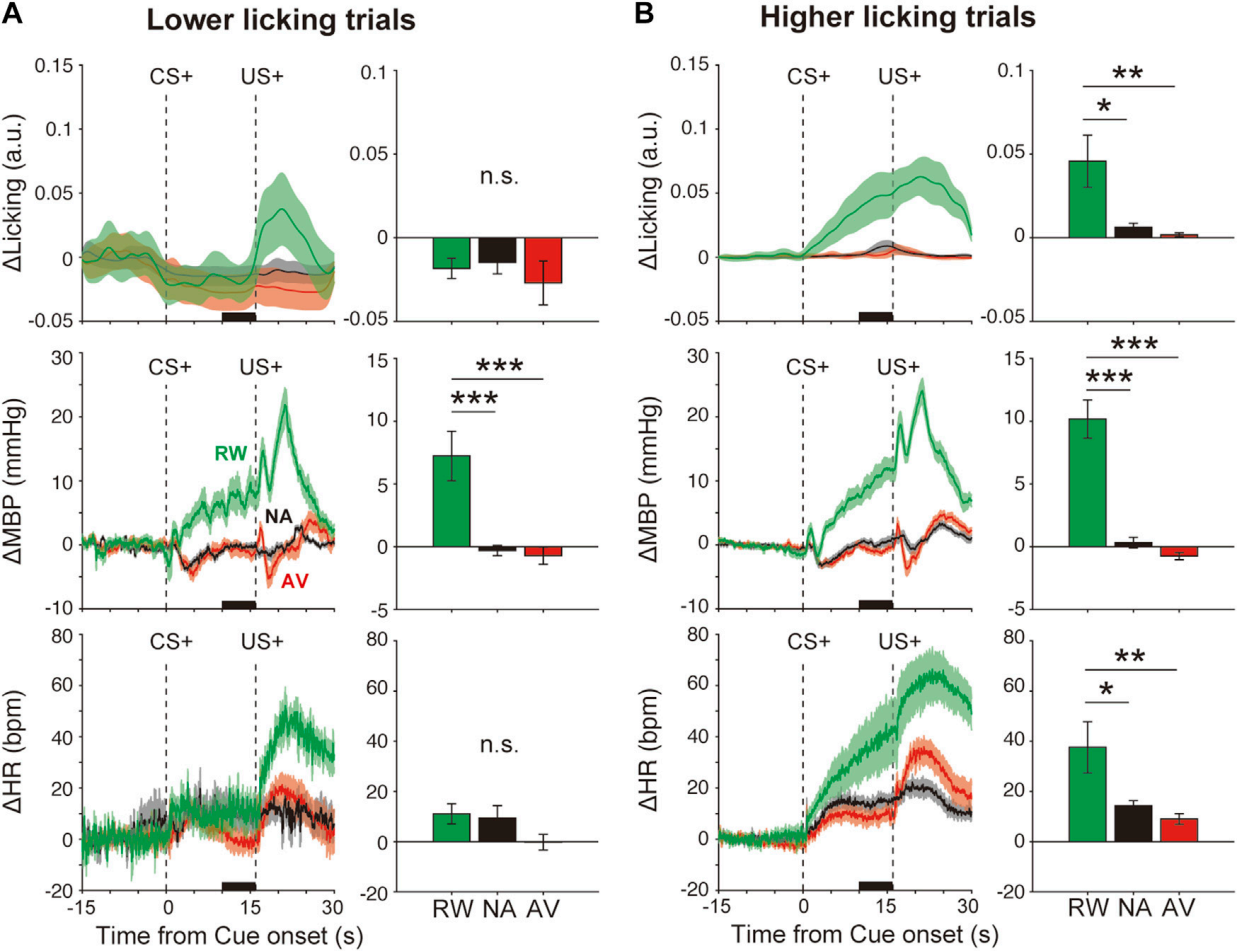
Effects of orofacial licking movements on cardiovascular responses. Average responses and quantitative bar graphs (inset of each figure) of licking (ΔLicking; top panels), mean blood pressure (ΔMBP; middle panels), heart rate (ΔHR; bottom panels), and responses to CS+ (left panels) and CS− (right panels) during the reward (RW; green line and bar), neutral (NA; black line and bar), and aversive (AV; red line and bar) condition blocks in trials with a **(A)** lower-licking group or **(B)** higher-licking group than the threshold (two standard deviations from the baseline) during the CS–US interval. *p < 0.05, **p < 0.01, ***p < 0.001, one-way analysis of variance with a Tukey–Kramer post hoc test, and n = 10 rats.

**FIGURE 4:**
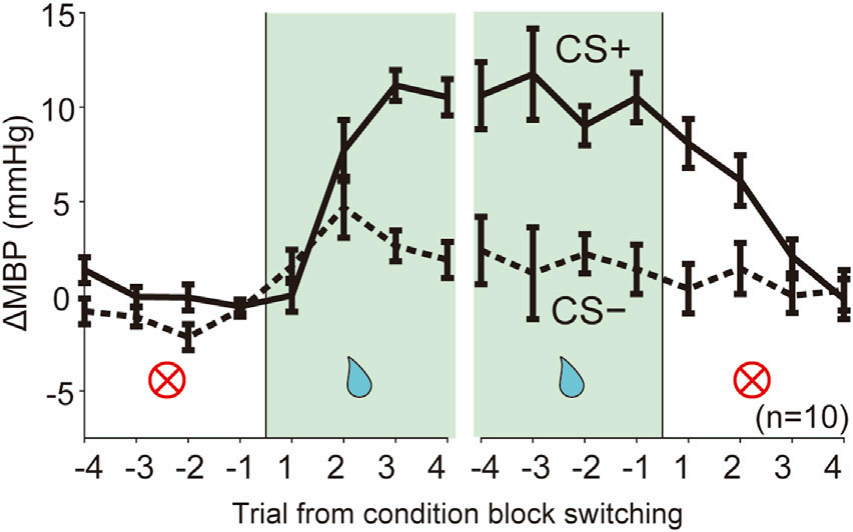
Dynamic changes of blood pressure responses triggered by context block switching. Line plots showing averaged ΔMBP during four trials in each trial type CS (solid line, CS+; dashed line: CS−) before and after switching of the reward condition block (NA→RW, RW→NA). RW, reward block; NA, neutral block. n = 10 rats.

**FIGURE 5:**
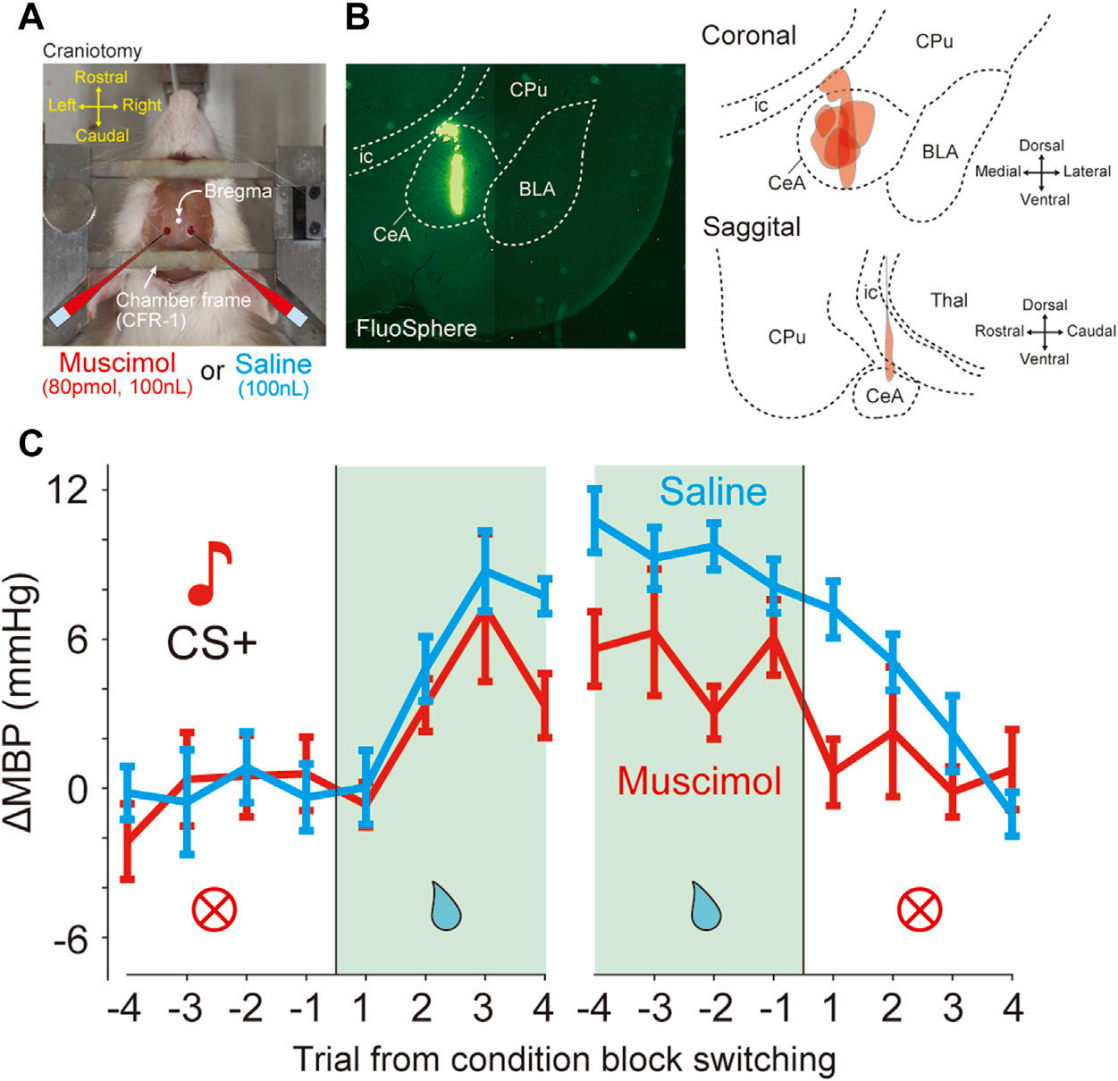
Bilateral inactivation of CeA attenuated the pressor response induced by reward prediction **(A)** Photograph shows a view of a head-restraint rat from above during drug injection. Craniotomy just above the amygdala (1.8 mm caudal, 3.0 mm lateral from bregma) is performed for drug injection **(B)** Injection site of six rats in the bilateral CeA. Fluorospheres (100 nL) were injected at identical stereotaxic positions using glass micropipettes (left panel). Reconstruction of the injection site in the coronal and sagittal planes (right panels). BLA, basolateral amygdala; CeA, central nucleus of the amygdala; CPu, caudate putamen; ic, internal capsule; Thal, thalamus. **(C)** Effects of bilateral inactivation in the data averaged from the mean blood pressure (ΔMBP) in response to CS + before and after four trials at condition block switching during the dynamically changing classical conditioning tasks. Red and cyan lines indicate the data obtained with muscimol and saline injections (six rats).

The blood pressure and heart rate were recorded simultaneously during the classical conditioning tasks using the telemetry blood pressure recording system (PhysioTel, Data Sciences International, United States) with the PowerLab system (PowerLab/8s, ADInstruments, New Zealand). The mean blood pressure and heart rate were derived from pulsatile pressure signals using LabChart software (Version 8.0, AD Instruments). The data were subsequently analyzed in MATLAB (The MathWorks, United States). Artificial changes in the blood pressure (<50 mmHg) and heart rate (<200 bpm or >600 bpm) signals were removed and treated as missing values in the dataset. We mainly analyzed the blood pressure and heart rate between the CS onset and the US onset (CS–US interval). Changes in the mean blood pressure (ΔMean blood pressure; ΔMBP) and heart rate (ΔHeart rate; ΔHR) were calculated by subtracting mean values during the baseline period 5–15 s before the CS onset. One-way analysis of variance (ANOVA) with the Tukey–Kramer post hoc test was used to compare the magnitudes of the blood pressure and heart rate measured 6 s immediately before the US onset among RW, NA, and AV blocks (Figure 2). Considering that the autonomic cardiovascular response is influenced by movement and emotion, we attempted to distinguish whether the observed cardiovascular responses were caused by orofacial movements due to the anticipatory licking behavior and/or by emotions induced by the outcome prediction. We divided the trials into two groups based on whether the licking amplitude was lower or higher than the threshold level (three standard deviations [SD] from the baseline) during the CS–US interval. The licking amplitude was converted into absolute values and the change from the baseline value was calculated for each trial (ΔLicking). Averaged blood pressure, heart rate, and licking movements were plotted and compared for low- and high-licking groups using one-way ANOVA with the Tukey–Kramer post hoc test (Figure 3). Statistical comparisons of the blood pressure were performed using the averaged data for each CS in four trials before and after the block switch (one-way ANOVA with the Tukey–Kramer post hoc test, Figure 4). Finally, we quantified and compared the CS–US intervals between sessions with muscimol and saline injections to examine the effects of CeA inactivation. The average data of ΔMBP in response to CS+ with or without bilateral inactivation of the CeA before and after four trials at the condition block switch during the classical conditioning task were assessed. We then analyzed the data using two-way ANOVA and the post hoc Tukey–Kramer test with trials from the condition block change, and with drugs (muscimol vs. saline) as factors (Figure 5). Statistical analyses were conducted using the MATLAB Statistics and Machine Learning Toolbox (The MathWorks). The mean blood pressure was averaged and statistically tested across rats (Figures 2–4, n = 10; Figure 5, n = 6). The criterion for statistical significance was *p* ≤ 0.05.

## Results

### Blood Pressure and Heart Rate Responses During Classical Conditioning Tasks

Although the animal body weights tended to be higher at the beginning of the week (Monday: mean ± SD = 104 ± 7%, % body weight to that immediately before water restriction) than those at the end of the week (Friday: mean ± SD = 97 ± 5%), overall, the weights remained constant throughout the week. To examine the effects of the head fixation to the stereotaxic frame, baseline blood pressure and heart rates during the first minute (immediately after immobilization) and the last minute (several hours after immobilization) of the task were plotted for each session (Supplementary Figure S1A,B). The blood pressure (130–140 mmHg) and heart rate (450–500 bpm) were high at the beginning of the training but decreased gradually as the training progressed to reach blood pressure (120–130 mmHg) and heart rate (350–400 bpm) values consistent with those from previous studies on classical conditioning tasks using reward and punishment in free-moving rodents (Shabel and Janak, 2009). In addition, absolute values of blood pressure and heart rate at the baseline (5–15 s before CS onset) for each trial after learning were approximately constant between 100 and 120 mmHg and between 300 and 350 bpm, respectively (Supplementary Figure S1C,D), during several hours of the experiment within a session. These results suggest that the immobilization did not overly stress the animals after habituation. Therefore, we subsequently analyzed data obtained after sufficient training. The data of the results of cardiovascular responses during the dynamically changing classical conditioning tasks (Supplementary Figure S2, 3, 4, Supplementary Tables S1, S2) were acquired from the session (day) 12 of the learning AV block, while the data of the results of inactivation experiments (Supplementary Figure S5, 3, 4, 5, 6, Supplementary Tables S3) were obtained afterward.

The averaged ΔMBP measured in response to the reward CS+ (+9.1 ± 1.4 mmHg) was significantly higher than that measured with the neutral CS+ (+0.4 ± 0.3 mmHg), *F*_(2,27)_ = 37.7, *p* < 0.001, and one-way ANOVA with the Tukey–Kramer post hoc test (Figure 2A and Supplementary Figure S2). The averaged ΔMBP triggered by aversive CS+ (−0.6 ± 0.3 mmHg) showed a tendency to decrease compared with that measured with neutral CS+, but was not significant (*p* > 0.05). Similarly, ΔHR in response to reward CS+ (33.3 ± 9.4 bpm) was significantly higher than that obtained in the NA block (12.9 ± 2.2 bpm), *F*_(2,27)_ = 6.1, *p* = 0.007, and one-way ANOVA with the Tukey–Kramer post hoc test (Figure 2B and Supplementary Figure S2). ΔHR induced by aversive CS+ (7.4 ± 1.9 bpm) showed a tendency to decrease compared with that of the neutral CS+ (*p* > 0.05). Although CS− (4 kHz tone) had no outcome in any trial throughout the classical conditioning tasks, ΔMBP, but not ΔHR (Figure 2D and Supplementary Figure S2, *F*_(2,27)_ = 1.5, *p* = 0.24), was significantly affected by reward CS− (ΔMBP, Figure 2C and Supplementary Figure S2, *F*_(2,27)_ = 5.5, *p* = 0.01).

We evidenced cardiovascular responses associated with reward prediction. These might reflect the emotional responses induced by positive outcome-associated cues. However, these responses might be caused by other events such as anticipatory licking movements as we observed that some animals displayed high anticipatory licking, whereas others did fewer anticipatory licking movements (Supplementary Figure S2). To examine this possibility, we divided the data into two groups, lower and higher licking groups, compared with the threshold value (2 SD from the baseline), according to the licking movement amplitudes during the CS–US interval (Supplementary Table S1). Even in the low-licking group (Figure 3A, top panel, *F*_(2,26)_ = 2.2, *p* > 0.05), ΔMBP induced by reward CS+ (+7.2 ± 2.0 mmHg) was significantly higher than that observed after neutral CS+ (−0.3 ± 0.4 mmHg) (Figure 3A, middle-left panel), *F*_(2,26)_ = 14.5, *p* < 0.001, and one-way ANOVA with a Tukey–Kramer post hoc test). However, conditioned heart rate responses disappeared in the lower licking trials (Figure 3A bottom panel), RW versus NA, *p* = 0.13; *F*_(2,26)_ = 2.2; and one-way ANOVA with Tukey–Kramer post hoc test. Conversely, in the higher licking trials (Figure 3B top panel, *F*_(2,27)_ = 7.0, *p* = 0.003), ΔMBP (*F*_(2,27)_ = 42.4, *p* < 0.001) and ΔHR (*F*_(2,27)_ = 6.2, *p* = 0.006) were increased proportionally to the licking amplitude (Figure 3B middle and bottom panels), suggesting that licking movements strongly affected the cardiovascular responses, especially the heart rate. Therefore, at least for the blood pressure, it is possible that the condition-dependent responses were caused by the licking movements and by the emotional cue–outcome association.

### Dynamic Changes in Cardiovascular Responses Triggered by the Context Block Switch

We assessed the predictive cardiovascular responses during condition block switching using dynamically changing appetitive and aversive classical conditioning tasks. The line plots of the averaged ΔMBP in response to CS+ and CS− in four trials before and after the block switch (Figure 4; NA→RW→NA, NA→AV→NA) indicate that the unanticipated switch from neutral to reward condition blocks (Figure 4 and Supplementary Table S2, NA→RW) in the first trial resulted in ΔMBP triggered by reward CS+ (0.05 ± 0.87 mmHg, *p* = 1.000; one-way ANOVA with a Tukey–Kramer post hoc test, compared with ΔMBP induced by CS+ in the NA block) similar to those obtained in the previous NA condition block. However, responses to reward CS+ in the second (7.71 ± 1.61 mmHg, *p* = 0.075) and third (11.15 ± 0.82 mmHg, *p* < 0.001) trials were dramatically increased after the rats had been exposed to reward US+. Pressor responses to reward CS+ were maintained for a few trials after switching back to the neutral block (RW→NA): 8.08 ± 1.30 mmHg in the first trial (*p* = 0.047), 6.13 ± 1.34 mmHg in the second trial (*p* = 0.355), and 2.11 ± 0.91 mmHg in the third trial (*p* = 0.999) after block switching. Therefore, cardiovascular responses to reward-predictive cues rapidly changed, guided by reward expectations.

### Bilateral Inactivation of the CeA Attenuated Reward Prediction-Induced Pressor Response

The GABA_A_ receptor agonist muscimol or saline was microinjected immediately before the conditioning task (Figure 5A). In total, we performed microinjection experiments on seven rats. All animals were injected with both muscimol and saline in different sessions. The injection site was verified by histology using fluorescent microspheres (Figure 5B). Because it was possible that one of the seven rats might not have been injected with CeA, the following analysis was performed on the data of six animals excluding it. The effects of injections on each animal were averaged and analyzed statistically (muscimol injections, n = 6 and saline injections, n = 6). In the dynamically changing classical conditioning task, compared with saline, muscimol microinjection of CeA caused significant decreases in ΔMBP before and after switching from the RW blocks (Figure 5C and Supplementary Table S3A), the main effect for drug factor, *F*_(1,160)_ = 7.47, *p* = 0.007; the main effect for trial factor, *F*_(15,160)_ = 4.47, *p* < 0.001; no interaction between drug and trial factors *F*_(15,160)_ = 0.62, *p* = 0.86, and two-way ANOVA. On the other hand, there was no significant difference in ΔHR (Supplementary Figure S3A; main effect for drug factor, F_(1,160)_ = 2.54, *p* = 0.11) and the licking behavior (Supplementary Figure S3B; main effect for drug factor, F_(1,160)_ = 1.02, *p* = 0.31) between muscimol and saline-injected animals as measured by the behavioral task.

In addition, we examined the effect on the baseline blood pressure after muscimol injection of CeA. However, there were no significant effects (Supplementary Figure S4; ΔMBP, time of injection, *p* = 0.80; drug (saline and muscimol), *p* = 0.10; ΔHR, time from injection, *p* = 0.39; drug (saline and muscimol), *p* = 0.53) or interactions (ΔMBP, time x drug, *p* = 0.89; ΔHR, time x drug, *p* = 0.68; two-way ANOVA).

A previous study reported that the effect of muscimol lasts for up to 3 h (Scheel-Kruger et al., 1977). In the muscimol injection experiments, the average time between the muscimol injection and the end of the task for each session in a day was 198 ± 43 min, which roughly corresponded to the duration of muscimol action. Moreover, we analyzed datasets obtained within 3 h after injection and observed similar effects, namely, a significant attenuation of the pressor response in the reward block (Supplementary Figure S5A and Supplementary Table S3B, main effect for drug factor, *F*_(1,160)_ = 7.19, *p* = 0.008, main effect for trial factor, *F*_(15,160)_ = 4.43, *p* < 0.001, no interaction between drug and trial factors *F*_(15,160)_ = 0.77, *p* = 0.71, two-way ANOVA). When the analysis was limited and datasets were obtained within 2 h after injection, similar results were observed (Supplementary Figure S5B and Supplementary Table S3C, main effect for drug factor, *F*_(1,160)_ = 10.06, *p* = 0.0018, main effect for trial factor, *F*_(15,160)_ = 3.36, *p* < 0.0001, no interaction between drug and trial factors, *F*_(15,160)_ = 0.53, *p* = 0.92, two-way ANOVA). In addition, to examine the sequential effects of muscimol and saline injections, we analyzed the effects of drugs on blood pressure responses before and after the reward block switching in separate datasets in all six rats: 1) a dataset including a muscimol injection session followed by a saline injection session (“muscimol→saline” dataset), and 2) another dataset including a saline injection session followed by a muscimol injection session (“saline→muscimol” dataset). Consistent with our results as shown previously, a significant main effect of the drug was observed in both injection datasets (Supplementary Figure S5C, “muscimol→saline,” *F*_(1,160)_ = 5.08, *p* = 0.025; Supplementary Figure S5D, “saline→muscimol,” *F*_(1,160)_ = 13.94, *p* = 0.0003).

Furthermore, we examined the effects of CeA inactivation on blood pressure responses to the actual outcome, US+. CeA inactivation by the muscimol injection showed a decreasing trend in US+ response (0–5 s after US+ onset), but the effect was not significant on the main effects for drug factors (Supplementary Figure S6, *F*_(1,160)_ = 2.98, *p* = 0.086, two-way ANOVA).

## Discussion

The present study showed that reward prediction increased blood pressure. Switching condition blocks provoked a rapid regulation of the blood pressure. Furthermore, blocking the activity of CeA impaired the pressor responses induced by reward condition block. We demonstrated that cardiovascular responses were adaptively and rapidly regulated by positive emotional stimuli and CeA might have contributed to the adaptive regulation of the blood pressure.

A phasic and gradual increase of the blood pressure was observed in response to reward-predictive cues. The blood pressure responses were maintained in lower-licking trials. Both the blood pressure and heart rate increased with the licking movement amplitude, suggesting that they were affected by orofacial licking movements including changes in muscle activity and breathing. However, at least the blood pressure might be regulated by additional factors such as emotion.

Previous studies have reported cardiovascular responses during classical conditioning tasks using free-moving and restrained animals from several species, such as pigeons (Cohen and Durkovic, 1966), rabbits (Pascoe and Kapp, 1985; Powell et al., 1997), dogs (Dykman and Gantt, 1956; Obrist and Webb, 1967; Anderson and Brady, 1972), rodents (Holdstock and Schwartzbaum, 1965; Iwata and LeDoux, 1988; Shabel and Janak, 2009; Shabel et al., 2011), marmosets (Braesicke et al., 2005; Mikheenko et al., 2010), macaques (Randall et al., 1975), and humans (Hastings and Obrist, 1967; Wexler and De Leon, 1979). Appetitive conditioning using food or water as a reward US, induces pressor response and tachycardia during the CS–US interval, whereas, in aversive conditioning, using electrical shocks as punishment US often causes pressor response or tachycardia, although some bradycardia responses have been reported (Harris and Brady, 1974; Cohen and Obrist, 1975; Randall et al., 1975). Here, a small decrease in the blood pressure was induced by air-puff predicting CS+. Based on the remarkable cardiovascular responses, air puffs acted as the US (Figure 2 and Supplementary Figure S2). Air-puff predicting CS + might have caused a slight suppression, and not a facilitation, of the cardiovascular responses because of the overtraining of animals, which could not avoid the punishment (e.g., learned helplessness), in the classical conditioning procedures. The lower response levels of aversive CS+ is that the air puff has been caused by the mildness of the stimulus compared with electrical shock and/or by a floor effect. In the present study, we used a neutral block in which CS+ was associated with no outcome as a different measure from the reward block. Because the activity of midbrain dopaminergic neurons encoding reward-prediction error signals decreases in no-reward-prediction cues (Matsumoto and Hikosaka, 2009), no outcome conditions might function as an aversive, rather than a neutral measure.

The roles of the amygdala in classical conditioning have already been examined, particularly in fear conditioning of free-moving animals. CeA has anatomical connections with various regions, such as the lateral hypothalamus, midbrain periaqueductal gray, locus coeruleus, and brainstem, to regulate autonomic and endocrine functions to promote defensive behaviors (such as attack, escape, and freeze) (Davis, 1992). Bilateral lesions to the amygdala attenuate responses associated with sympathetic excitation (pressor, tachycardia, and sympathetic cutaneous vasomotor alerting responses) induced by appetitive (Braesicke et al., 2005) and aversive (Iwata et al., 1986; LeDoux et al., 1990; Mikheenko et al., 2010) conditioning and unconditioning salient stimuli (Mohammed et al., 2013) in free-moving animals. These findings suggest that the amygdala is involved in autonomic arousal in emotional processing. Several studies have examined either appetitive or aversive condition blocks; however, little is known concerning autonomic tuning during dynamical alternations between appetitive and aversive conditions. Furthermore, it has been shown that disinhibition of CeA using bicuculline induced pressor and tachycardiac responses under anesthesia (Yamanaka et al., 2018), and the blockage of the amygdala by muscimol induced acute cutaneous vasodilatations and impaired sympathetic cutaneous vasoconstrictor alerting-responses under natural unconditioning situations (Mohammed et al., 2013). Our results are consistent with the previous findings that show that the amygdala (CeA) plays an important role in accelerating the blood pressure in response to salient stimuli (Iwata et al., 1986; LeDoux et al., 1990; Braesicke et al., 2005; Mikheenko et al., 2010; Mohammed et al., 2013).

The lack of significant changes in the cardiovascular responses to aversive-predicting cues activated by the amygdala is a limitation in the present study. This result might be caused by the imbalanced impact of the outcomes between the reward and relatively mild air puff, even though air puffs (air jets) are used as aversive stimuli (Knapp and Pohorecky, 1995; Belova et al., 2007; Cohen et al., 2012; Furlong et al., 2014). In addition, repeated air-puff stimulation has also been reported to cause habituation (Knapp and Pohorecky, 1995). To solve these problems, it is necessary to focus on earlier periods before habituation occurs, or by using different types of aversive stimuli such as electrical shock. There were several problems with the pharmacological method in this study that were required to manipulate the neuronal activity, which might have resulted in a non-specific manipulation of neuronal circuits, variations in the injection site accuracy by diffusion of the drugs, and low temporal resolution of neuronal manipulation. Whether the attenuation of the predictive-pressor response elicited by CeA inactivation is caused by forgetting of the associative memory or by the inhibition of the autonomic drive based on memory although memory remains, is not clear in this study. To solve these problems, future studies are necessary to examine CeA-specific genetic manipulation using optogenetics and/or chemogenetics.

Our findings showed cardiovascular regulation and the involvement of CeA in dynamically changing CS–US associations. However, CeA is not the only contributor to the formation of predictive circulatory responses. The characteristic blood pressure responses rapidly (after a few trials) adapt immediately after condition block switching. Such a rapid switch is reminiscent of the activity of neurons of the amygdala (Belova et al., 2007), striatum (Lauwereyns et al., 2002), and of midbrain dopaminergic neurons (Roesch et al., 2007). Blockade of the dopamine D1 receptor in the striatum impairs context-dependent behavioral responses (Nakamura and Hikosaka, 2006; Ueda et al., 2017). The artificial inhibition of midbrain dopaminergic neurons during the fear-to-safety context switch induced a delay in the extinction of fear-conditioning behavior (Luo et al., 2018). In addition, dopaminergic neuron activity has also been reported to contribute to the prediction of reward and aversion stimuli (Matsumoto and Hikosaka, 2009; Moriya et al., 2018). Anatomically, the amygdala receives dopaminergic inputs (Garris and Wightman, 1995). There is a direct projection from the amygdala (basolateral region) to the striatum, whereas the projection from the striatum to the amygdala is believed to be polysynaptic. The activities of striatal neurons increase in response to reward-predicting cues, whereas they are attenuated by the blockade of the amygdala (Ambroggi et al., 2008). Therefore, the condition-dependent cardiovascular responses observed in the present study might reflect the differences in the outcome expectation, which are dependent on the functional interactions between the amygdala and striatum via dopaminergic inputs (Averbeck and Costa, 2017). Future studies should examine the functions of neuronal circuits potentially supporting and regulating the emotional expression suitable to a dynamically changing environment.

## Data Availability

The raw data supporting the conclusion of this article will be made available by the authors, without undue reservation.
