# Supplementary material for: Conditional Regulation of Blood Pressure in Response to Emotional Stimuli by the Central Nucleus of the Amygdala in Rats
Source: Front Physiol. 2022 Jun 1;13:820112. doi: 10.3389/fphys.2022.820112 (PMC9198497; doi:10.3389/fphys.2022.820112)
Supplement: Supplementary file 1 [file DataSheet1.pdf]

Supplement Figures and Table

Figure S1. Variations in the absolute values of blood pressure and heart rate.

(A-B) Average (mean ± standard error) blood pressure (A) and heart rate (B) measured during the first minute (magenta line, First 1 min) after the task started and the first minute (cyan line, Last 1min) after task ended were plotted for each session.

(C-D) Averaged (mean ± standard error) absolute values of blood pressure (C) and heart rate (D) at baseline (5-15 s before CS onset) for each trial within one session before (session 1, dotted line and gray shadow) and after learning (session 12, solid line and dark gray shadow).

Figure S2. Blood pressure, heart rate, and licking responses of each animal (n = 10) during the dynamically changing classical conditioning task.

Average responses of ΔMBP, ΔHR, and ΔLicking to CS+ and US+ and to CS– and US– during the reward (RW; green line and bar), neutral (NA; black line and bar), and aversive (AV; red line and bar) condition blocks in each rat (Rat#1~#10). Lines and shadows indicate mean ± standard error.

Figure S3. Effects of bilateral CeA inactivation by muscimol injections on ΔHR (A) and the anticipatory licking behavior (B) during dynamically changing classical conditioning tasks.

Effects of bilateral inactivation to the heart rate (A, ΔHR) and anticipatory licking (B, ΔLicking) in response to CS+ before and after 4 trials at condition block switching in the reward block during the dynamically changing classical conditioning task. Red and cyan lines indicate the data obtained with muscimol and saline injections (6 rats). Black dotted line indicates the data obtained with no injection (10 rats).

Figure S4. Acute effects of muscimol and saline bilateral injections in the CeA on baseline blood pressure and heart rate.

Average (mean ± standard error) blood pressure (A) and heart rate (B) responses to bilateral injections of muscimol (red line) or saline (cyan line) into the CeA. Two-way analysis of variance of the effect of the time of injection (min) and drug (muscimol and saline), n = 6.

Figure S5. Effects of bilateral CeA inactivation by muscimol injection on blood pressure responses to CS+ during dynamically changing classical conditioning tasks.

(A-B) Same as Figure 5, but data were acquired within 180 min (A) and 120 min (B) of injection for each animal.

(C-D) A data set including a muscimol injection session followed by saline injection session (C, "muscimol→saline" data set), and another data set including a saline injection session followed by muscimol injection session (D, "saline→muscimol" data set).

Figure S6. Effects of bilateral CeA inactivation by muscimol injection on blood pressure responses to US+ during dynamically changing classical conditioning tasks.

Effects of bilateral inactivation in the data averaged from the mean blood pressure (ΔMBP) in response to US+ before and after 4 trials at condition block switching during the dynamically changing classical conditioning task. Red and cyan lines indicate the data obtained with muscimol and saline injections (6 rats). No significant main effect on drug was observed. Two-way ANOVA.

Table S1. Ratio of lower licking trials and higher licking trials in each CS.

Table S2. Averaged ΔMBP during 4 trials in each trial type CSs (solid line, CS+; dashed line: CS−) before and after switching of reward condition block (NA→RW, RW→NA). The data expressed as means ± SD (n = 10). The p-value in the ΔMBP responses of CS+ indicates the result compared to the -1 trial in the NA block before the RW block (one-way ANOVA with post hoc Tucky-Kramer test). No significant main effect was observed in the ΔMBP responses of CS−. *: $p < 0.05$; **: $p < 0.01$; ***: $p < 0.001$. RW, reward block; NA, neutral block.

Table S3. Effects of bilateral CeA inactivation on blood pressure responses to CS+ during a dynamically changing classical conditioning task.

The data expressed as means ± SD (n = 6). Data were acquired within all trials (A), 180 min (B) and 120 min (C) of muscimol injection for each animal.

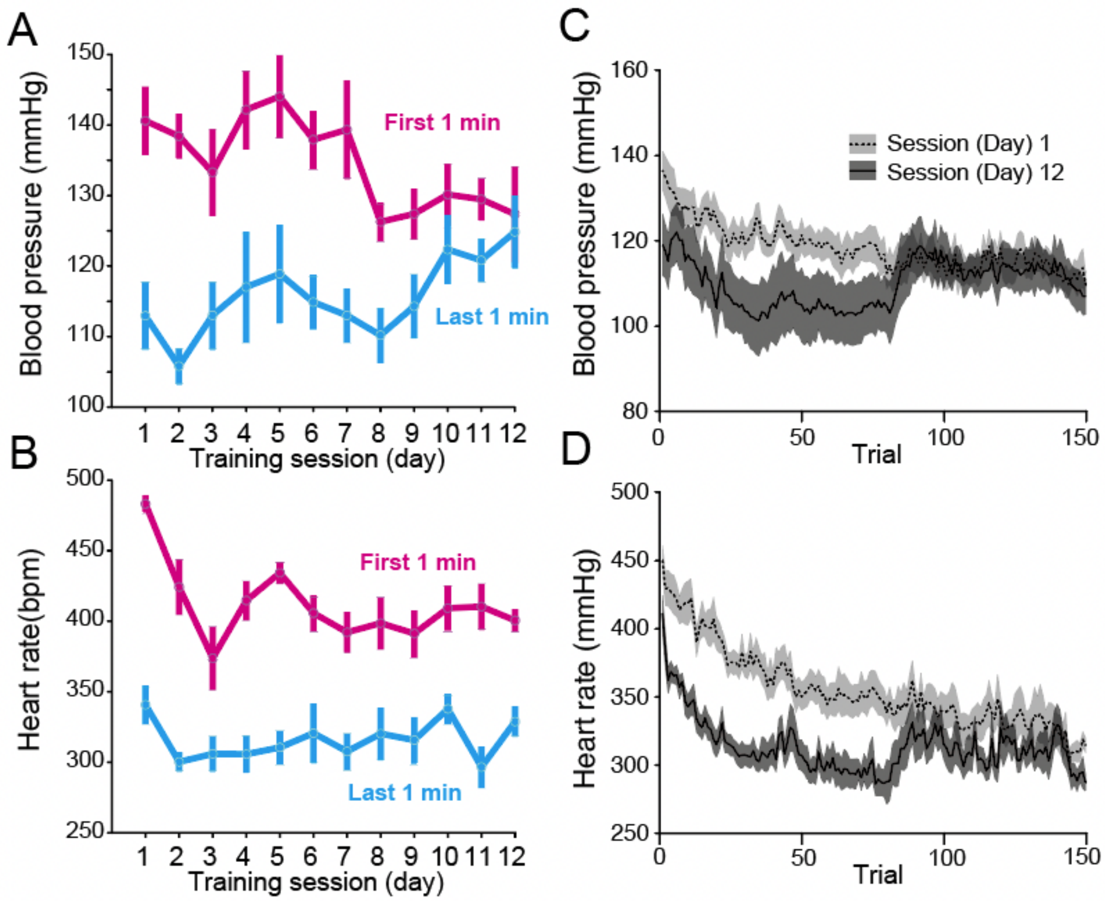

A
Blood pressure (mmHg)
150
140
130
120
110
100
First 1 min
Last 1 min
1 2 3 4 5 6 7 8 9 10 11 12
Training session (day)
B
Heart rate(bpm)
500
450
400
350
300
250
First 1 min
Last 1 min
1 2 3 4 5 6 7 8 9 10 11 12
Training session (day)
C
Blood pressure (mmHg)
160
140
120
100
80
Session (Day) 1
Session (Day) 12
0
50
100
150
Trial
D
Heart rate (mmHg)
500
450
400
350
300
250
0
50
100
150
Trial

Figure S1

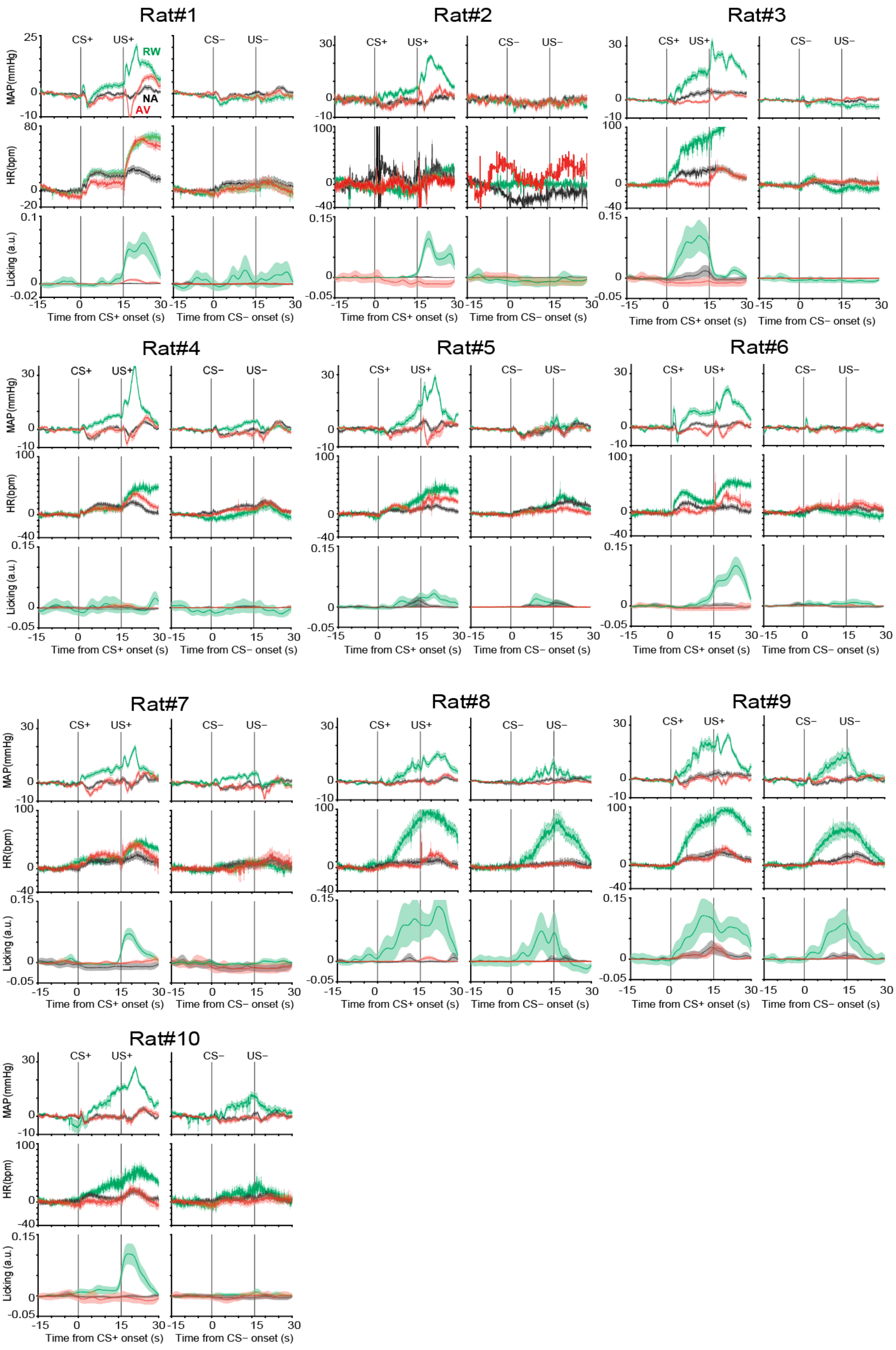
Rat#1
Rat#2
Rat#3
Rat#4
Rat#5
Rat#6
Rat#7
Rat#8
Rat#9
Rat#10
RW
NA
AV
CS+
US+
CS−
US−
MAP(mmHg)
HR(bpm)
Licking (a.u.)
Time from CS+ onset (s)
Time from CS− onset (s)

Figure S2

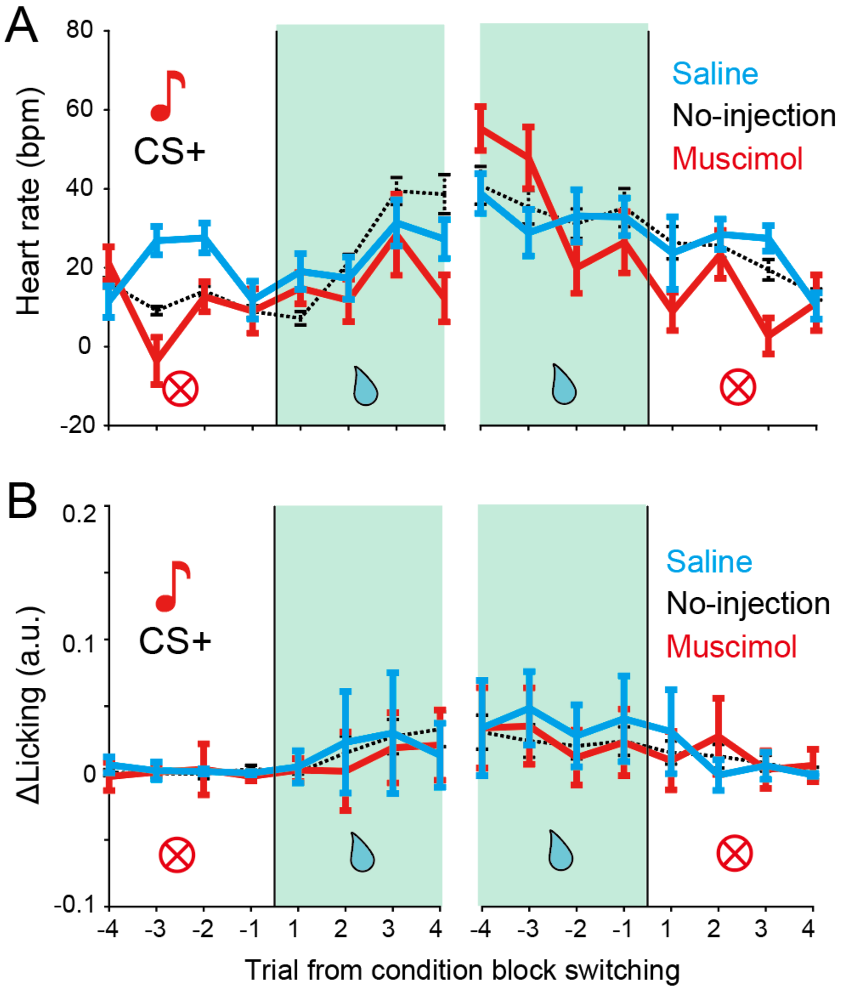
A
B
Heart rate (bpm)
ΔLicking (a.u.)
80
60
40
20
0
-20
0.2
0.1
0
-0.1
CS+
CS+
Saline
No-injection
Muscimol
Saline
No-injection
Muscimol
-4 -3 -2 -1 1 2 3 4 -4 -3 -2 -1 1 2 3 4
Trial from condition block switching

Figure S3

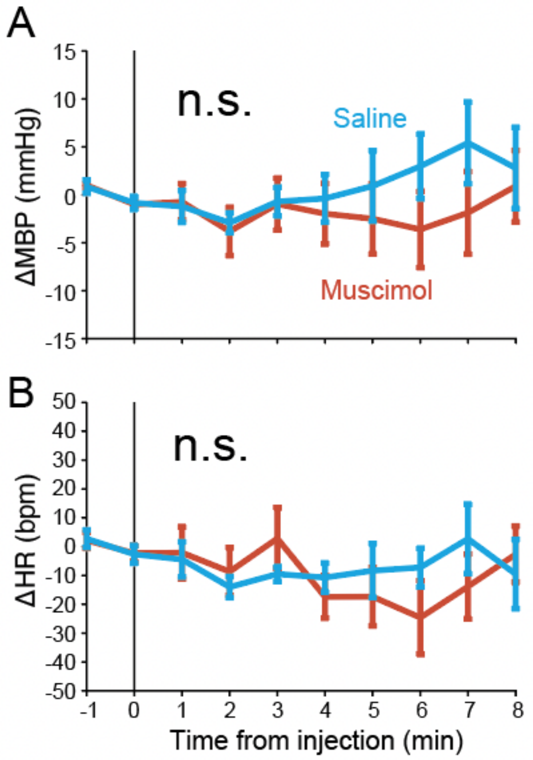
A
ΔMBP (mmHg)
15
10
5
0
-5
-10
-15
n.s.
Saline
Muscimol
B
ΔHR (bpm)
50
40
30
20
10
0
-10
-20
-30
-40
-50
n.s.
-1
0
1
2
3
4
5
6
7
8
Time from injection (min)

Figure S4

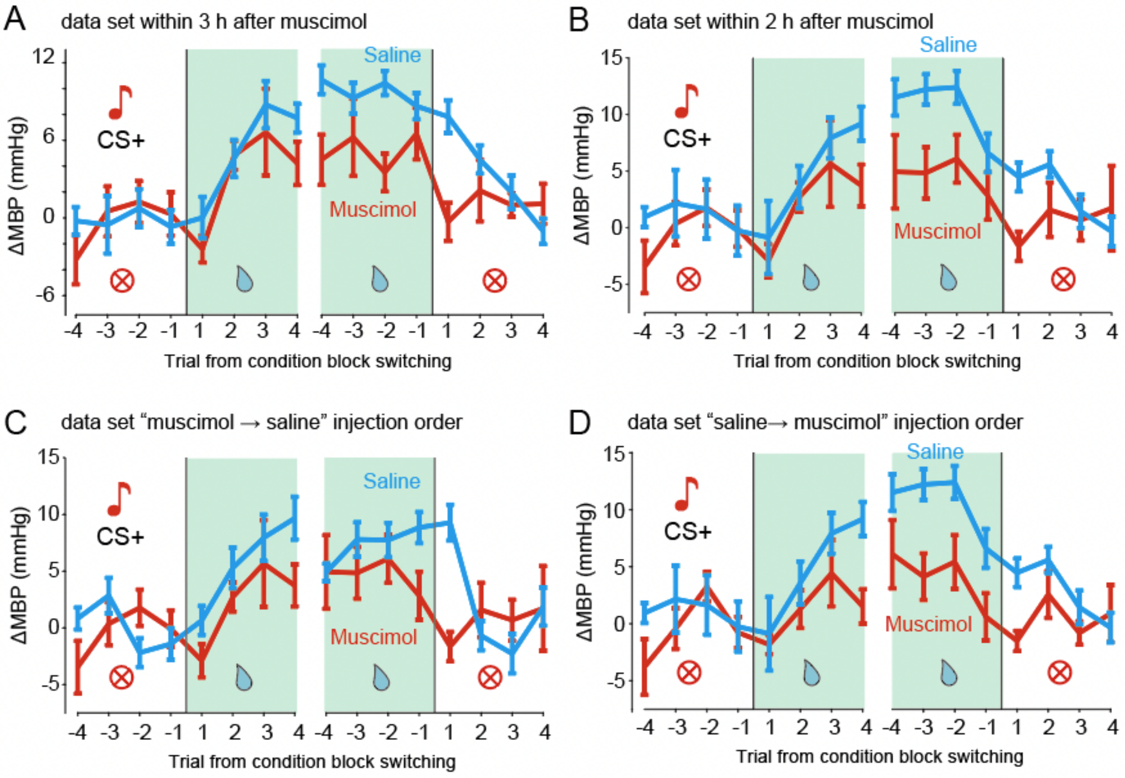
A
data set within 3 h after muscimol
B
data set within 2 h after muscimol
C
data set "muscimol → saline" injection order
D
data set "saline→ muscimol" injection order
ΔMBP (mmHg)
CS+
Saline
Muscimol
Trial from condition block switching

Figure S5

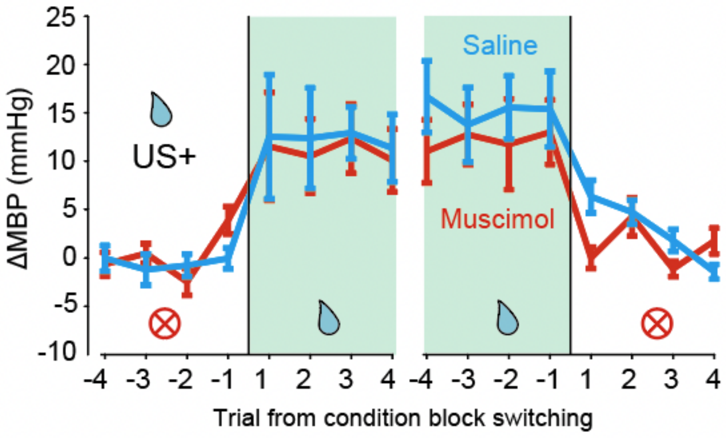
25
20
15
10
5
0
-5
-10
ΔMBP (mmHg)
US+
Saline
Muscimol
-4 -3 -2 -1 1 2 3 4 -4 -3 -2 -1 1 2 3 4
Trial from condition block switching

Figure S6

Table S1

	Rat#1		Rat#2		Rat#3		Rat#4		Rat#5	
	Low(%)	High(%)	Low(%)	High(%)	Low(%)	High(%)	Low(%)	High(%)	Low(%)	High(%)
CS+(RW)	60.0	40.0	65.5	34.5	9.7	90.3	37.9	62.1	57.7	42.3
CS-(RW)	72.5	27.5	77.4	22.6	97.0	3.0	48.6	51.4	66.7	33.3
CS+(NA)	80.0	20.0	94.4	5.6	75.4	24.6	92.9	7.1	79.5	20.5
CS-(NA)	83.3	16.7	83.1	16.9	98.2	1.8	8.8	91.2	80.5	19.5
CS+(AV)	90.0	10.0	93.8	6.3	93.9	6.1	85.7	14.3	89.5	10.5
CS-(AV)	76.7	23.3	93.8	6.3	96.8	3.2	96.4	3.6	76.2	23.8

	Rat#6		Rat#7		Rat#8		Rat#9		Rat#10	
	Low(%)	High(%)	Low(%)	High(%)	Low(%)	High(%)	Low(%)	High(%)	Low(%)	High(%)
CS+(RW)	56.0	44.0	67.7	32.3	25.0	75.0	9.5	90.5	34.8	65.2
CS-(RW)	73.7	26.3	71.0	29.0	15.0	85.0	21.1	78.9	47.1	52.9
CS+(NA)	57.5	42.5	66.7	33.3	57.7	42.3	48.4	51.6	86.7	13.3
CS-(NA)	67.6	32.4	72.1	27.9	66.7	33.3	77.4	22.6	71.9	28.1
CS+(AV)	90.0	10.0	81.0	19.0	63.6	36.4	45.5	54.5	81.8	18.2
CS-(AV)	80.0	20.0	78.9	21.1	78.3	21.7	77.3	22.7	77.3	22.7

Table S2

Blood pressure (mmHg)										
		CS+					CS-			
	trial	Mean		SD	*p*		Mean		SD	
NA	-4	1.39	±	0.68	-		-0.81	±	0.70	
	-3	-0.02	±	0.51	-		-1.10	±	0.50	
	-2	-0.06	±	0.69	-		-2.18	±	0.75	
	-1	-0.54	±	0.36	-		-0.73	±	0.74	
RW	1	0.05	±	0.87	1.000		1.56	±	1.44	
	2	7.71	±	1.61	0.075		4.66	±	1.09	
	3	11.15	±	0.82	<0.001	***	2.63	±	1.24	
	4	10.53	±	0.96	0.001	**	1.89	±	1.18	
RW	-4	10.61	±	1.78	<0.001	***	2.39	±	1.15	
	-3	11.75	±	2.42	0.001	**	1.20	±	1.15	
	-2	9.02	±	1.04	0.012	*	2.21	±	1.46	
	-1	10.51	±	1.30	0.001	**	1.40	±	1.72	
NA	1	8.08	±	1.30	0.047	*	0.38	±	0.60	
	2	6.13	±	1.34	0.355		1.44	±	1.27	
	3	2.11	±	0.91	0.999		0.02	±	1.27	
	4	-0.14	±	1.06	1.000		0.29	±	1.05	

p value: vs. NA(-1) trial

Table S3

A. All data

	trial	Muscimol Mean		Muscimol SD	Saline Mean		Saline SD
		Blood pressure (mmHg)					
NA	-4	-2.14	±	1.52	-0.19	±	1.06
	-3	0.36	±	1.88	-0.55	±	2.10
	-2	0.49	±	1.64	0.84	±	1.42
	-1	0.59	±	1.48	-0.37	±	1.35
RW	1	-0.66	±	0.90	0.04	±	1.49
	2	3.35	±	1.06	4.81	±	1.30
	3	7.27	±	2.97	8.74	±	1.62
	4	3.33	±	1.29	7.73	±	0.70
RW	-4	5.61	±	1.50	10.77	±	1.27
	-3	6.28	±	2.54	9.26	±	1.23
	-2	3.05	±	1.06	9.74	±	0.93
	-1	6.08	±	1.52	8.14	±	1.07
NA	1	0.64	±	1.34	7.20	±	1.14
	2	2.27	±	2.62	5.08	±	1.13
	3	-0.15	±	1.00	2.21	±	1.52
	4	0.76	±	1.61	-1.04	±	0.89

B. within 180 min

	trial	Muscimol Mean		Muscimol SD	Saline Mean		Saline SD
		Blood pressure (mmHg)					
NA	-4	-3.24	±	1.89	-0.23	±	1.06
	-3	0.52	±	1.93	-0.53	±	2.23
	-2	1.25	±	1.62	0.76	±	1.46
	-1	0.30	±	1.68	-0.69	±	1.30
RW	1	-2.41	±	1.04	0.02	±	1.60
	2	4.83	±	1.11	4.60	±	1.43
	3	6.63	±	3.35	8.76	±	1.82
	4	4.22	±	1.67	7.72	±	1.12
RW	-4	4.51	±	1.94	10.68	±	1.11
	-3	6.24	±	2.98	9.26	±	1.19
	-2	3.53	±	1.46	10.43	±	0.93
	-1	6.52	±	1.99	8.64	±	1.01
NA	1	-0.30	±	1.48	7.83	±	1.27
	2	2.12	±	2.39	4.53	±	1.08
	3	1.00	±	0.90	1.98	±	1.29
	4	1.09	±	1.56	-1.02	±	0.98

C. within 120 min

	trial	Muscimol Mean		Muscimol SD	Saline Mean		Saline SD
		Blood pressure (mmHg)					
NA	-4	-3.46	±	2.32	0.94	±	0.87
	-3	0.36	±	1.91	2.16	±	2.94
	-2	1.76	±	1.60	1.64	±	2.61
	-1	-0.08	±	1.61	-0.26	±	2.21
RW	1	-2.90	±	1.47	-0.87	±	3.23
	2	2.70	±	1.29	3.58	±	1.87
	3	5.66	±	3.82	7.92	±	1.80
	4	3.72	±	1.85	9.18	±	1.50
RW	-4	4.94	±	3.25	11.51	±	1.61
	-3	4.84	±	2.28	12.21	±	1.35
	-2	6.09	±	2.12	12.39	±	1.44
	-1	2.82	±	2.11	6.61	±	1.71
NA	1	-1.65	±	1.29	4.48	±	1.27
	2	1.57	±	2.40	5.59	±	1.16
	3	0.68	±	1.81	1.44	±	1.48
	4	1.72	±	3.74	-0.34	±	1.30
